# Alpha7 nicotinic acetylcholine receptor is required for blood-brain barrier injury-related CNS disorders caused by *Cryptococcus neoformans* and HIV-1 associated comorbidity factors

**DOI:** 10.1186/s12879-015-1075-9

**Published:** 2015-08-19

**Authors:** Bao Zhang, Jing-Yi Yu, Li-Qun Liu, Liang Peng, Feng Chi, Chun-Hua Wu, Ambrose Jong, Shi-Fu Wang, Hong Cao, Sheng-He Huang

**Affiliations:** Department of Microbiology, Guangdong Provincial Key Laboratory of Tropical Disease Research, School of Public Health and Tropical Medicine, Southern Medical University, Guangzhou, 510515 China; Saban Research Institute of Childrens Hospital Los Angeles, Department of Pediatrics, University of Southern California, 4650 Sunset Blvd., Mailstop #51, Los Angeles, CA 90027 USA; Division of Pediatric Neurology, Children’s Medical Center, The Second Xiangya Hospital of Central South University, Changsha, Hunan 410011 China; Department of Clinic Laboratory, the Second Affiliated Hospital of Guangzhou Medical University, Guangzhou, 510260 China; Department of Children’s Medical Laboratory Diagnosis Center, Qilu Children’s Hospital of Shandong University, Jinan, 250022 China

## Abstract

**Background:**

Cryptococcal meningitis is the most common fungal infection of the central nervous system (CNS) in HIV/AIDS. HIV-1 virotoxins (e.g., gp41) are able to induce disorders of the blood-brain barrier (BBB), which mainly consists of BMEC. Our recent study suggests that α7 nAChR is an essential regulator of inflammation, which contributes to regulation of NF-κB signaling, neuroinflammation and BBB disorders caused by microbial (e.g., HIV-1 gp120) and non-microbial [e.g., methamphetamine (METH)] factors. However, the underlying mechanisms for multiple comorbidities are unclear.

**Methods:**

In this report, an aggravating role of α7 nAChR in host defense against CNS disorders caused by these comorbidities was demonstrated by chemical [inhibitor: methyllycaconitine (MLA)] and genetic (α7^−/−^ mice) blockages of α7 nAChR.

**Results:**

As shown in our *in vivo* studies, BBB injury was significantly reduced in α7^−/−^ mice infected with *C. neoformans*. Stimulation by the gp41 ectodomain peptide (gp41-I90) and METH was abolished in the α7^−/−^ animals. *C. neoformans* and gp41-I90 could activate NF-κB. Gp41-I90- and METH-induced monocyte transmigration and senescence were significantly inhibited by MLA and CAPE (caffeic acid phenethyl ester, an NF-κB inhibitor).

**Conclusions:**

Collectively, our data suggest that α7 nAChR plays a detrimental role in the host defense against *C. neoformans*- and HIV-1 associated comorbidity factors-induced BBB injury and CNS disorders.

## Background

Cryptococcal meningitis is the most common fungal infection of the central nervous system (CNS) and also the most prevailing neurological complication in AIDS patients: AIDS patients often develop severe neuro-degeneration and dementia, which is also habitually aggravated by other ailments such as methamphetamine (METH) abuse. Among them, the patients commonly succumb to the co-infected yeast opportunistic pathogen *Cryptococcus neoformans.* The mortality rate of HIV-associated cryptococcal meningitis is high (~30 %), and is one of the major causes of death in AIDS patients [1]. In order to cause meningoencephalitis, *C. neoformans* cells must cross the blood-brain barrier (BBB). The BBB is constituted by the brain microvascular endothelial cells (BMEC), distinguished by their tight junctions amongst cells [2]. We have previously studied the invasion of *C. neoformans* with the human BMEC (HBMEC) using *in vitro* binding and transcytosis assays [3, 4]. *C. neoformans* induces considerable morphological changes and actin reorganization on HBMEC [3]. Some molecular events have been observed for the formation of the *C. neoformans* entry site, including: the roles of CD44 [5, 6], caveolin-1 [7], and PKCα [8] on the membrane lipid rafts, and the role of endocytic kinase DYRK3 during the *C. neoformans* internalization [9]. Perceivably, *C. neoformans* implements complicated invasion mechanisms inherent to this pathogen.

The interrelationship between HIV-1 and *C. neoformans* is intriguing, as both pathogens elicit severe neuropathological complications. We have previously demonstrated that the HIV-1 gp41 enhances *C. neoformans* binding to HBMEC *in vitro* and increase *C. neoformans* brain invasion *in vivo* [10]. We have further elucidated the underlying mechanism by showing that both *C. neoformans* and gp41-I90 up-regulate ICAM-1 on the HBMEC, cause the redistribution of CD44 and β-actin on the lipid rafts, and induce membrane ruffling on the surface of HBMEC [6, 11]. These results suggest that the HIV-1 gp41-I90 enhances *C. neoformans* binding with HBMEC via gp41-I90-induced membrane activities, revealing a potential mechanism for this pathogenic fungus to invade the brain tissues of HIV-1-infected patients. The progression of HIV disease and its consequences may be worsened by METH abuse. METH is able has been shown to increase viral replication in animal models. METH abusers with HIV have shown greater chance to have neuronal injury and cognitive impairment, compared with the HIV patients who do not abuse the drug. Many studies have demonstrated that METH, nicotine and microbial factors (e.g., gp41 and gp120) were able to significantly cause functional and structural changes in BBB [3, 12–16]. It also raised an interesting question: what is the gp41- and METH-induced host signaling to create such microenvironment that is favorable to the *C. neoformans* infection. As such, we can observe the enhanced infection both *in vitro* and *in vivo* [10, 11].

Alpha7 nicotinic acetylcholine receptor (α7 nAChR) is a major cholinergic ligand-gated ion channel in the CNS, has been implicated in the modulation of the BBB integrity and pathogenesis of CNS disorders caused by microbial (e.g., HIV-1 virotoxins gp120) and non-microbial (e.g., METH and nicotine) factors [17–19]. Alpha7 nAChR also contributes to the pathogenesis of Alzeimer’s disease (AD) [20]. It seems that α7 nAChR could be potential therapeutic target for HIV-1 and related comorbid factors-induced pathogenicities. Understanding the role of α7 nAChR in HIV-1 virotoxins- and related comorbid factors-induced pathogenicities will lead to improvements in diagnosis, prevention, and treatment of NeuroAIDS. Our recent studies have shown that the α7 nAChR cholinergic pathway is essential for lipid raft (LR)-dependent Ca2+ signal transduction, which leads to NF-κB activation, CNS inflammation and leukocyte transmigration across the BBB [21–23]. In this report, we have hypothesized that α7 nAChR -mediated signaling is a common pathway of CNS disorders caused by *C. neoformans,* HIV-1 virotoxins (gp41) and METH. We used both the *in vitro* (HBMEC) and *in vivo* (α7 nAChR knockout mice) models of the BBB to test the hypothesis mentioned above.

## Methods

### Chemicals and reagent

Evans blue, methamphetamine (METH) and MLA were purchased from Sigma-Aldrich (St. Louis, MO). Dynabeads M-450 Tosylactivated was purchased from Invitrogen (Carlsbad, CA). Ulex europaeus I (UEA I) lectin and mounting medium with DAPI were purchased from Vector (Buringame, CA). The Gp41 ectodomain peptide (gp41-I90) was prepared as described previously [9, 11]. A senescence β-Galactosidase Staining Kit (Cat#9860) was bought from Cell Signaling Technology. A Fluoro-Jade B (FJB) staining kit was obtained from Histo-Chemo Inc. All primary antibodies (Ab) were purchased from the commercial sources: a rabbit anti-α7 nAChR Ab from Genescript (Piscataway, NJ); Anti-NF-κB/p65 mAb from Cell Signal Technology (Cell Signal Technology, #3033); an antibody against dimethyl-histone H3 (Lys9) from Millipore (Millipore, cat #.07–212), an anti-mouse CD146 Ab FITC-conjugated and a mouse anti-neuron (NeuN) Ab from eBiosciences (San Diego, CA); a mouse anti-CD44 Ab (sc-7297); a rabbit anti-CD54 Ab (ICAM-1, 250593) from Abbiotec (San Diego, CA); a rabbit anti-β-actin (sc-7210) and an anti-GFAP Ab from Santa Cruz Biotechnology (Santa Cruz, CA); an anti-mouse CD146 Ab FITC-conjugated from Biolegend (San Diego, CA), and a rabbit anti-S100B Ab from BD Biosciences.

### Strains, media and cultures

B-4500FO2 is a wildtype strain of *C. neoformans* strain [4–6]. Yeast cells were grown aerobically at 30 °C in 1 % yeast extract, 2 % peptone and 2 % dextrose (YPD broth) or Sabouraud medium (Difco Laboratories, Detroit, MI). Cells were harvested at an early log phase, washed with phosphate-buffered saline (PBS) and then resuspended in Hams-F12 / M199 (1:1, v:v), 5 % heat-inactivated fetal bovine serum (experimental medium), and 1 % human serum. The Cryptococcus cell number was determined by direct counting from a hemocytometer.

### Animal model and treatment protocol

Heterozygous (**+/−**) α7-deficient mice with the C57BL/6 J background (B6.129S7-Chrna7tm1Bay/J) were purchased from Jackson Laboratory (Bar Harbor, ME). Genotypes of α7 +/+ mice (WT mice), α7**−/−** mice (KO mice) and heterozygous α7 +/− mice were determined according to the PCR protocol provided by the vendor. The animals were used in transgenic breeding at 8 weeks of age for optimum reproductive performance. Male heterozygous (+/−) and female homozygous (**−/−**) were used in breeding. The average litter size for neonatal mice was 6–8. Age- and sex-matched mice were used in all experiments. All experiments were approved by the Animal Care and Use Committee of Childrens Hospital Los Angeles Saban Research Institute. Two sets of experiments were performed. In the first experiment, the mice (6 week-old) were divided into 4 groups (I: WT + METH; II: WT + PBS; III: KO + METH; IV: KO + PBS). Two groups (I and III) of animals were treated with gradually increased doses (2, 4, 6, 8, 10, 10, 10, 10, 10, 10 mg/kg from day1 to day10) of METH [intraperitoneal (i.p.) injection] for 10 days as described previously [15]. The animals were infected with a total of 10^6^ yeast cells via lateral tail vein injection. After 24 h, the brain was removed, washed with 2 ml PBS. The tissues were homogenized and plate onto triplicate YPD plates for CFU counting. The presence of Cryptococcus cells in CSF is an indicator of cryptococcal meningitis, which was determined as described previously [4–6]. Briefly, C57LB/6 WT and KO mice were infected with yeast cells as described above. After 24 h, the animal was perfused with 20 ml PBS to remove blood circulating yeast cells. The skull was opened for CSF collection. The brain was then removed and washed with 500 μl PBS. In the meantime, the cranial cavity was washed with 100 μl PBS four times. The washing solutions were combined. After centrifugation, the pellet was resuspended into 50 μL PBS, designated as the CSF fraction. An aliquot was used for counting erythrocytes (RBC). Samples were discarded if the CSF was contaminated with blood >25 RBCs/μl present in the sample. The uncontaminated samples were used for Cryptococcus cell counting. In the second experiment, the animals were divided into 4 groups (I: WT + gp41-I90 + METH; II: WT + gp41-I90; III: KO+ gp41-I90 + METH; IV: KO+ gp41-I90). Two groups (I and III) of animals were treated with METH as described in the first experiment. The animals in Group I-IV received daily injections from tail veins of endotoxin-free recombinant HIV-1 gp41-I90 as described previously [11]. Mice were infected with yeast cells as described above.

### Isolation and purification of mouse brain microvascular endothelial cells

Mouse CEC and cBMEC were isolated from blood and brain tissues of the animals in the second experiment with Ulex europaeus I (UEA I) lectin-coated Dynabeads as described previously [15]. The beads were prepared according to the manufacturer’s instructions (Invitrogen) and resuspended in Hanks' balanced salt solution (HBSS, Invitrogen Corp., Carlsbad, CA, USA) plus 5 % fetal calf serum (HBSS + 5 %FCS) to a final concentration of 4 × l0^8^ beads/ml. The CEC and cBMEC were prepared as described previously [15]. Briefly, endothelial cells from blood were isolated by absorption to Ulex-coated beads. The cells were positive for CD146 [15], demonstrating their endothelial origin, and also expressed S100B [24], indicating their brain origin. For the cBMEC assays, the cells were transferred to glass splices to by cytospin for staining and counting under a fluorescence microscope. Total ECs or CECs (CD146+/DAPI+) and cBMECs (CD146+/S100B+/DAPI+) were identified based on their S100B [24] (brain marker)+/CD146 (EC marker)+/DAPI (nuclei) + phenotypes.

### Monocytes transmigration assay

Monocytes suspensions in experimental medium were used for transmigration assays as described previously [25]. Briefly, the confluence of the HBMECs monolayers in transwell filters (3.0 μm pore size, 6.5 mm diameter, BD Biosciences) coated with collagen was confirmed by light microscopy before the start of the assay. To test effects of inhibitors α7 nAChR (MLA), and NF-κB (CAPE) on gp41- and METH-induced monocyte transmigration across HBMECs. The HBMECs monolayers were pre-incubated with different doses of MLA (0, 0.1 μM, 1 μM, 10 μM) for 1 h or CAPE (0, 1 μM, 5 μM, 25 μM) for 2 h and stimulated with gp41 (10 μg/ml) and METH(50 nM) in the upper chambers. Then, Freshly isolated monocyte-like vitamin D3-differentiated HL60 cells (1x10^6^ cells/ml) were added to the upper chamber and allowed to migrate over for 4 h. The MLA or CAPE was present throughout the monocytes transmigration experiment until the end. At the end of the incubation, migrated HL60 cells were collected from the lower chamber and counted in a blinded-fashion using a hemacytometer. Final results of monocytes transmigration were expressed as the percentage of HL60 cells across the HBMECs monolayers.

### Immunoblotting analysis

To assess *C. neoformans*- and gp41-innduced NF-κB activation in HBMECs, endothelial cell monolayers were grown on 60-mm plates. Confluent HBMEC monolayers were incubated with *C. neoformans* (10^5^/ml), or gp41-I90 (1 μg/ml) for different time points. After the completion of incubation, cytoplasmic and nuclear proteins of HBMECs were extracted with lysis buffer supplied with 100 nM okadaic acid and 1 mM Na_3_VO_4_ as described previously [26]. Both cytoplasmic and nuclear proteins were mixed with SDS buffer, heated and subjected to sodium dodecyl sulfate polyacrylamide gel electrophoresis (SDS–PAGE). Separated proteins were transferred to nitrocellulose membrane by semi-dry blotting. After blocking with 5 % milk in PBS with 0.1 % Tween 20 for 2 h, cytoplasmic and nuclear proteins were probed with antibodies against phospho-NF-κB/p65 (ser536) and β-actin overnight, respectively. The washed membranes were incubated with a horseradish peroxidase (HRP)-conjugated secondary antibody for 1 h and then visualized using an enhanced chemiluminescence procedure (Roche Applied Science, Indianapolis, IN).

### Analysis of HBMECs and astrocytes senescence induced by *C. neoformans* and gp41-I90

The detection of senescence phenotypes of HBMEC and astrocytes was according to the kit procedure of Cell Signaling Technology. Briefly, after the treatment with 1 μg gp41-I90 for 24 h, HBMECs were rinsed with PBS one time. Cells were fixed with 1 × fixative solution for 10–15 min at room temperature prior to be stained with β-Galactosidase staining solution at 37 °C for at least 3 days. About 500 randomly selected cells per sample were photographed under a microscope (200× total magnification) with positive cells developing blue color. Senescence associated heterochromatin formation (SAHF) were detected with the Narita’s method [27]. Cells were treated same as β-Galactosidase activity detection, then stained with an antibody against dimethyl-histone H3 (Lys9) and DAPI.

### Histology staining of neural tissue

Mice brains were harvested for fluoro-Jade B (FJB) staining [28, 29] and immunostaining with an anti-NeuN antibody [30] after infection, fixed in 10 % buffered formalin for 24 h, embedded in paraffin, and sections with 5 μm thickness were prepared. Dried mounted brain sections were fixed with 4 % PFA vapors in a closed container placed in a water bath heated at 37 °C for 2.5 h. Slides were dehydrated and rehydrated through graded concentrations of alcohol (50, 70, 100, 70, and 50 % alcohol, 1 min each), and rinsed with distilled water for 1 min. Slides were then treated with potassium permanganate (0.06 %) for 10 min and rinsed for 1 min in distilled water, prior to be stained in 0.004 % FJB in 0.1 % acetic acid for 20 min. Finally, Slides were rinsed in distilled water (3 × 1 min), dried overnight at 37 °C, then imaged with flour-microscopy. To perform immunostaining, the tissue sections were stained with a FITC-conjugated mouse antibody against neuron-specific nuclear protein (NeuN) (eBiosciences), and counterstained with DAPI. Image fluorescence quantification analysis was performed with program MetaMorph (Version 7.7.3.0) and TUNEL assays. For each treatment, 5–6 mouse brains were sectioned and stained.

### Statistical analysis

The statistical analysis of the data from our study involved analysis of variance (ANOVA). The dependent variable was the associated percent of cells or CFU while the independent fixed factors were be the treatments (wildtype vs. KO). Raw data was entered into EXCEL files and automatically converted to the compatible format for statistical analysis packages. ANOVA and co-variates were followed by a multiple comparison test such as the Newmann-Keuls test to determine the statistical significance between the control and treatment groups. *P* < 0.05 was considered to be significant.

### Ethics statement

All research involving human participants has been approved by the Institutional Review Board (IRB) of Children’s Hospital Los Angeles (CHLA). Human brain microvascular endothelial cells (HBMEC) were isolated in accordance with the protocol approved by the CHLA Committee on Clinical Investigations (CCI), which is the IRB for Human Subjects at Saban Research Institute of CHLA. This protocol has been granted a waiver of informed or signed consent per 45 CFR 46.116(d) and a waiver of HIPAA authorization per the Privacy Rule (45 CFR Part 160 and Subparts A and E of Part 164). No minors/children participants were involved in our studies. The animal study was performed in strict accordance with the recommendations in the Guide for the Care and Use of Laboratory Animals of the National Institutes of Health. Our protocols were approved by the Institutional Animal Care and Use Committee (IACUC) of The Saban Research Institute of CHLA (Permit number: A3276-01). All surgery was performed under anesthesia with ketamine and lidocaine, and all efforts were made to minimize suffering.

## Results

### Alpha7 nAChR- and NF-κB-mediated signaling is required for monocyte transmigration across HBMEC

**Fig. 1:**
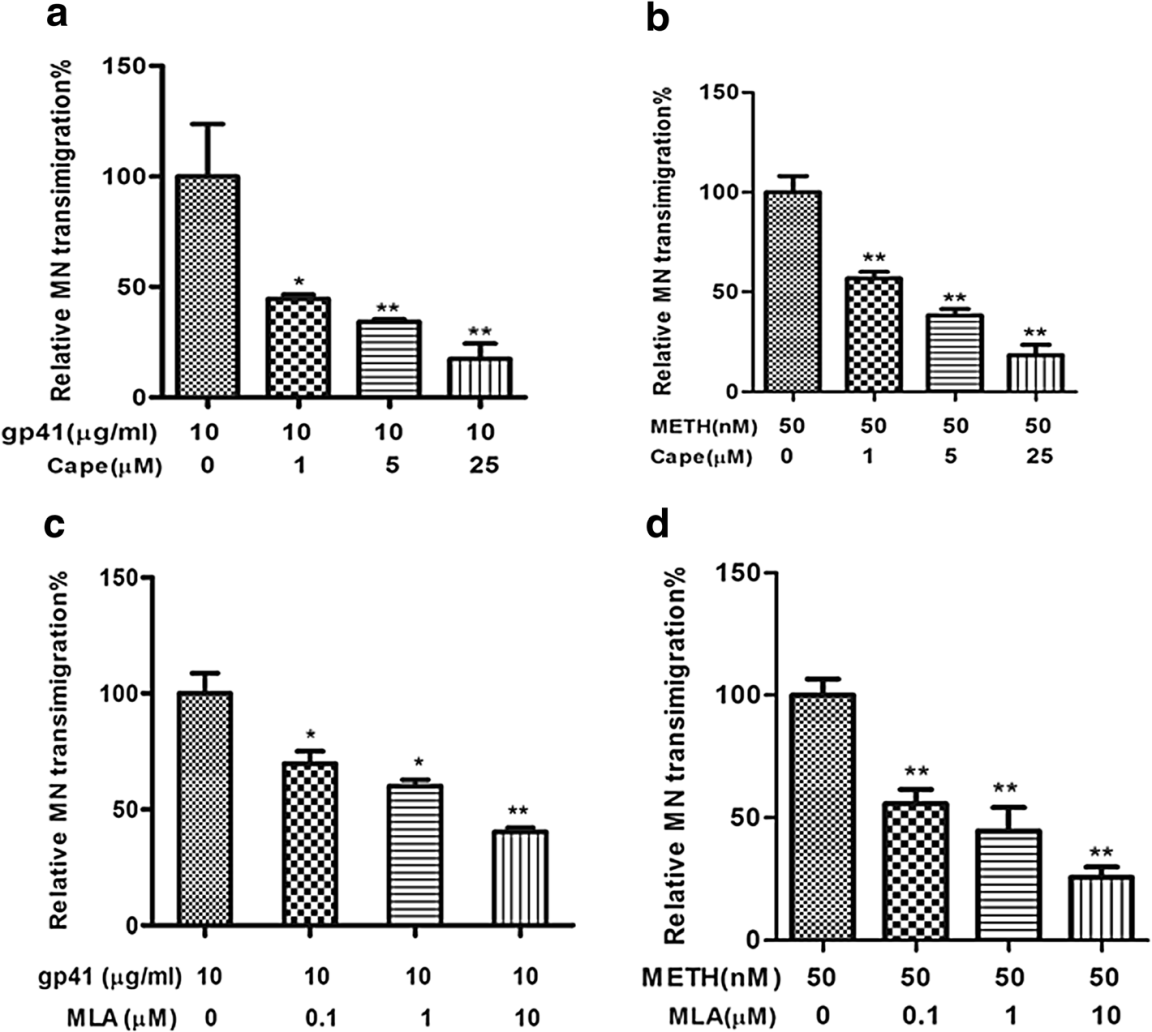
Effects of blockages of α7 nAChR and NF-κB on gp41-I90- and METH-induced monocyte transmigration across BBB. HBMECs were pre-incubated with or without gp41-I90 (10 μg/ml) and METH (50 nM) for 48 h, and then treated with different doses of inhibitors CAPE (0, 1 μM, 5 μM, 25 μM) for 2 h or MLA (0, 0.1 μM, 1 μM, 10 μM) for 1 h before the Monocytes transmigration assays. Freshly isolated monocyte-like vitamin D3-differentiated HL60 cells (1x10^6^ cells) were added to the upper chamber and allowed to migrate over for 4 h. (**a**-**b**) CAPE (NF-κB inhibitor) could dose-dependently block gp41- and METH-induced monocyte transmigration across HBMEC. (**c**-**d**) MLA (α7 antagonist) was able to inhibit gp41- and METH-induced monocyte transmigration across HBMEC in a dose-dependent manner. Values represent the means of relative transmigrating monocytes of triplicate samples. In (**a**-**d**), the experimental setting without inhibitor treatment was taken as a control (the first column). Bar graphs showed the means ± SD of the triplicate samples. *P < 0.05, **P < 0.01

Monocyte recruitment into the CNS plays a crucial role in the inflammatory response induced by HIV-1 and related comorbidity factors [31–33]. In order to exclude the possibility that the leukocyte migration elicited was due to destruction of HBMEC, the integrity of the monolayer was inspected by microscopy. HBMEC were exposed to METH (50 nM) and gp41-I90 (10 μg/ml) for 48 h, different doses of MLA (1–10 μM) for 1 h or CAPE (1–25 μM) for 2 h, and subjected to monocyte transmigration assays. As indicated in Fig. 1a-d, gp41-I90 and METH could significantly increase leukocyte transmigration. The inhibitors of NF-κB (CAPE) and α7 nAChR (MLA) were able to significantly inhibit monocyte transmigration across the HBMEC monolayer treated with gp41-I90 (Fig. 1a and c) and METH (Fig. 1b and d) in a dose-dependent manner. These results suggest that α7 nAChR- and NF-κB-mediated signaling is required for leukocyte transmigration across the blood-brain barrier. It is consistent with the prior reports that α7 nAChR is able to regulate the NF-κB signaling pathway [15, 34].

### Alpha7 knockout mice are defensive in *C. neoformans*-, METH- and gp41-I90- induced BBB injury

**Fig. 2:**
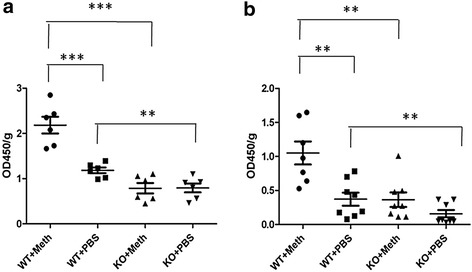
Effects of genetic blockage of α7 nAChR on *C. neoformans* (Cn)- and METH-increased BBB permeability. Mice were infected with Cn at 45 days (**a**) and 75 days (**b**). Evaluation of BBB permeability to Evans blue in Cn-infected WT and KO mice with or without METH exposure (*n* = 6–8). **P < 0.01, ***P < 0.001

**Fig. 3:**
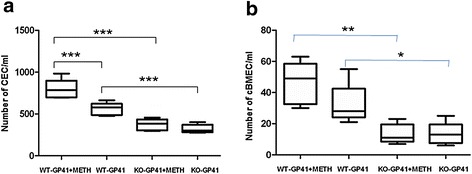
Effects of A7R KO on BBB disorders induced by gp41-I90 and METH. cBMECs were isolated from WT and A7R KO mice treated with gp41-I90 (GP41) or METH as described in our recent publications [9, 49]. Cells without treatment were used as a control. Triple staining (TS) was done by DAPI (blue)/antibodies against CD146 (FITC/green) and S100B (for brain) (rhodamine/red). **a**: CECs (CD146^+^/DAPI^+^), and **b**: cBMECs (CD146^+^/S100B^+^/DAPI^+^). Cells were counted with six random fields. (*P <0.05; **P < 0.01; ***P < 0.001)

To further validate the biological relevance of the *in vitro* assays, the role of α7 nAChR in the pathogenesis of CNS disorders induced by HIV-1 and related comorbidity factors was tested in the mouse model, as described in Methods and Materials. The host susceptibility to BBB injury induced by these pathogenic insults was tested in α7 nAChR wildtype (α7+/+) and KO (α7−/−) mice pretreated with METH or gp41-I90. Animals of the same age were injected through tail vein with *C. neoformans* (2x10^6^ CFU), followed by Evans blue (EB) injection after 15 h. As shown in Fig. 2a, EB was significantly decreased in the brain tissues of KO mice as compared to that of wildtype animals (*P* < 0.01), suggesting that α7 nAChR plays an important regulatory role in the BBB integrity. This result showed that the magnitude of EB was significantly increased by METH exposure only in wildtype mice (*P* < 0.001), but not in KO mice, suggesting that α7 nAChR is essential for METH-enhanced *C. neoformans*-induced pathogenicities (Fig. 2a-b). However, there is no significant age difference between the two groups (45 days in Fig. 2a vs 75 days in Fig. 2b) of animals. Similarly, as shown in the Fig. 3b, the cell-based BBB biomarker cBMEC in the blood was significantly reduced in KO mice as compared to wildtype animals (*P* < 0.01). The blood levels of CEC, which is the cell-based biomarker of peripheral vasculature, were remarkably decreased in KO mice (Fig. 3a), suggesting that α7 nAChR also contributes to the regulation of the peripheral vasculature integrity. Taken together, these data suggested that α7 nAChR could play a detrimental role in the host defense against *C. neoformans,* HIV-1 gp41 and METH.

### *C. neoformans*- and gp41-I90- induced cytoplasmic activation and nuclear translocation of NF-κB

**Fig. 4:**
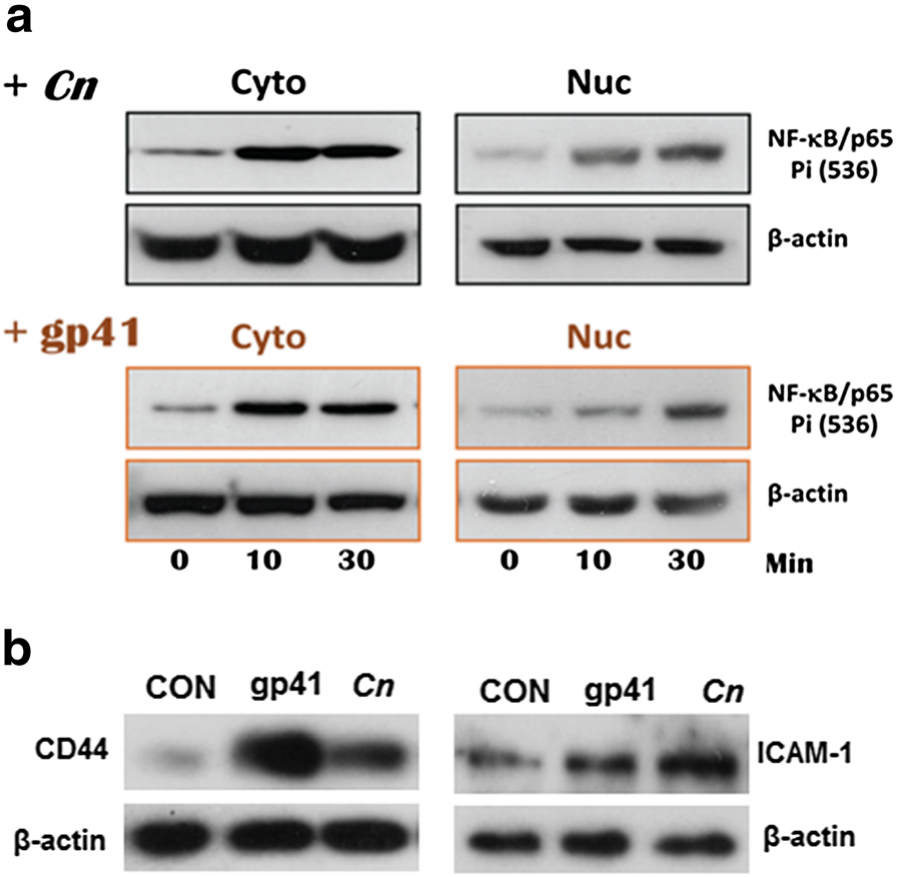
*C. neoformans* (Cn)- and gp41-I90- induced activation of NF-κB/p65 that resulted in up-regulation of CD44 and ICAM-1. (**a**) HBMEC were treated with either *Cn* cells (upper, 10^5^ cells/mL) or gp41-I90 (bottom, 1 μg/mL), and harvested at different time points as indicated. Cell pellets were fractionated into cytosol and nuclear fractions, and subjected to the Western blots. Cyto (30 μg) and Nuc (10 μg) per lane were loaded. An anti-NF-κB/p65 mAb was used to probe the phosphorylation site Ser(536) of NF-κB/p65. (**b**) Expression of CD44 and ICAM-1 were examined (*Cn* 10^7^ cells/mL or gp41-I90 20 μg/mL, 6 h), respectively, using antibodies against CD44 and ICAM-1. β-Actin was the loading control

As shown in our previous studies [11], gp41-I90 is able to significantly enhance *C. neoformans* meningitis. Because the mechanisms by which *C. neoformans* and gp41 induce signaling are still poorly understood and the initiation of NF-κB activation and subsequent nuclear translocation is not clear, we performed time course analysis of *C. neoformans*- and gp41-I90-induced NF-κB activation and translocation. HBMECs were treated with *C. neoformans*- and gp41-I90. The proteins from cytoplasmic and nuclear fractions were examined in Western blots using the antibody against the activated NF-κB/p65 (ser536). As shown in Fig. 4a, *C. neoformans* (the upper panel) and gp41-I90 (the lower panel) could increase cytoplasmic NF-κB activation and subsequent nuclear translocation in a time-dependent manner. NF-κB activation resulted in up-regulation of inflammatory factors CD44 and ICAM-1 (Fig. 4b). These findings suggest that NF-κB signaling is required for CNS disorders caused by *C. neoformans* and HIV-1 virulence factors.

### *C. neoformans*- and gp41-I90- induced Neuronal injury in the brain sections containing prefrontal cortex

**Fig. 5:**
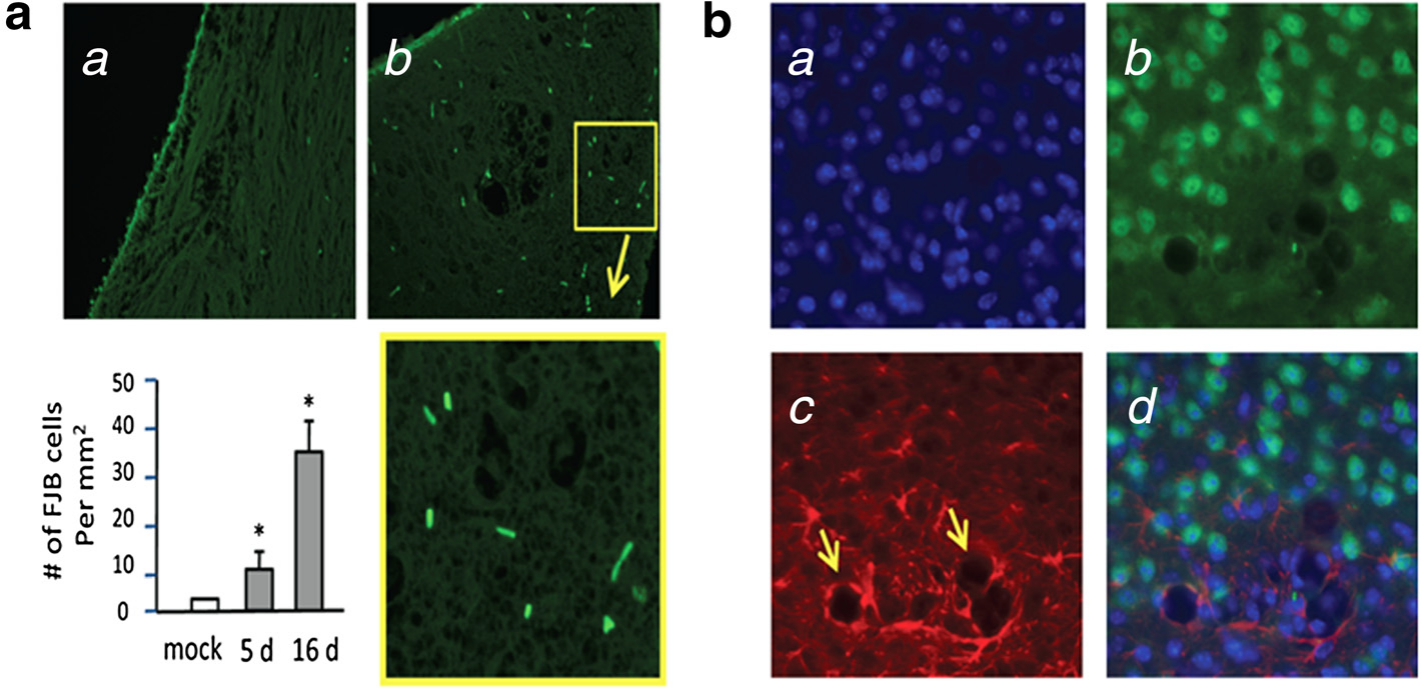
Neuronal damages of *Cn* infected brains that contain prefrontal cortex. (**a**) The FJB signals of the control (*a*) and *Cn*-infected mouse brain sections (5 day infection) (*b*) are shown. The yellow box is magnified shown in below. Mean number of FJB positive neurons/mm^2^ (*n* = 5) at 5- and 16- days are significantly different from the mock control (*P < 0.05). (*b*) A *Cn*-infected brain section shows (*a*) nuclear DNA (DAPI stain), (*b*) neuron cells (NeuN), (*c*) reactive astrocytes (GFAP), and (*d*) the overlaid image. The upper part of the image seems normal, but the lower part shows that the reactive astrocytes surrounding the cystic lesions (yellow arrows), where the NeuN signals disappear near the lesions. Similar observations were obtained from gp41-I90 treatment, but the signals were more dispersed through the whole brain section (data not shown)

Both HIV-1 and *C. neoformans* cause severe neuronal damages, and the age population is more vulnerable to these ailments [35]. The onset, process and interrelationship with pathogen insults relevant to the age are still not clear. We have used the neuronal markers, fluoro-Jade B (FJB) [28, 29] and NeuN [30], to examine the infected mouse brains. The enhancement of FJB signals indicates the increase of neuronal degeneration (Fig. 5a). On the other hand, the disappearance of the NeuN signals around the cystic lesion suggests the loss of neuron cells in the damaged area (Fig. 5b).

### HIV-1 gp41-I90- and comorbidity factors-induced senescence in HBMECs and astrocytes were blocked by the α7 nAChR antagonist MLA

**Fig. 6:**
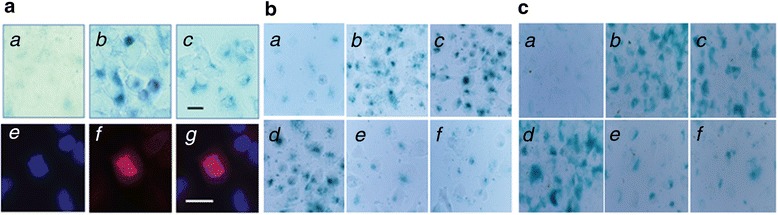
Senescence phenotypes of HBMECs and astrocytes are induced by HIV-1 related comorbidity factors and blocked by MLA. (**a**) SA-βgal images of HBMEC were obtained with the treatment of: (*a*) mock, (*b*) 1 μg gp41-I90 for 24 h, (*c*) *Cn* cells (10^4^ cells) in a chamber slide for 24 h. Blue spots in (*b*) & (*c*) indicate senescence cells. For SAHF images, HBMEC was treated with 1 μg gp41-I90 for 24 h, and stained with an antibody against dimethyl-histone H3 (Lys9). Immunofluorescence microscopic images detect (*e*) nuclear DNA (DAPI, blue), (*f*) heterochromatin foci =(SAHF, red), and (*g*) the overlaid image. Bar = 25 μm. (**b**) *a*: Control (HBMECs), *b*: gp41-I90 (1 μg/ml), *c*: METH (50 nM), *d*: gp41-I90 (1 μg/ml) + METH (50 nM), *e*: MLA+ gp41-I90 (1 μg/ml), *f*: MLA+ METH (50 nM). (**c**) *a*: Control (astrocytes), *b*: gp41-I90 (1 μg/ml), *c*: METH (50 nM), *d*: gp41-I90 (1 μg/ml) + METH (50 nM), *e*: MLA+ gp41-I90 (1 μg/ml), *f*: MLA+ METH (50 nM)

As the age population increases and HIV-1 infection increases in older population, the incidence of NeuroAIDS becomes higher and accelerated among the aging populations. Likewise, in *C. neoformans* infection, age ≥60 year-old is a significant predictor of the mortality [36, 37]. To test whether gp41 and comorbidity factors were able to induce senescence in HBMECs, which could be blocked by the α7 antagonist MLA, HBMECs were treated with *C. neoformans* and gp41-I90. Senescence associated heterochromatin formation (SAHF) were detected with the Narita’s method. β-Galactosidase activity was measured. The cells were stained with DAPI and an antibody against dimethyl-histone H3 (Lys9). These morphological changes and the increase in SA β-gal stained cells confirmed that HIV-1 gp41-I90 and *C. neoformans* efficiently induced the premature senescence of HBMECs (Fig. 6a). Next, the α7 antagonist MLA was used to illustrate the roles of α7 nAChR in gp41-I90 and its comorbid factor-induced premature senescence of HBMECs and astrocytes. We compared senescence of HBMECs and astrocytes upon exposure to gp41-I90, METH, METH + gp41-I90, and treatment with MLA 1 h before incubation with these stimulating agents. When treated with either 1 μg/ml gp41-I90, 50 nM METH or METH (50 nm) + gp41-I90 (1 μg/ml) (24 h), HBMECs and astrocytes became flat and showed enlarged morphology, which is a characteristic phenotypic change in premature senescence. MLA significantly reduced these morphological changes and the number of SA β-gal stained HBMEC (Fig. 6b) and astrocytes (Fig. 6c). These results suggest that α7 antagonist MLA suppresses HIV-1 gp41-I90 and comorbidity factors-induced senescence in HBMECs and astrocytes.

## Discussion

Currently, the mechanisms responsible for the pathogenesis of the CNS disorders caused by HIV-1 and related comorbidity factors remain unclear. An important connection between the nervous system and the inflammatory response to disease has been uncovered through identification of α7 nAChR, an ion channel highly permeable to calcium, as an essential regulator of inflammation [38–40]. Alterations of brain microvascular endothelial cells (BMEC), which form the BBB, and the pathogenesis of neurodegenerative disorders (ND), are commonly associated with HIV-1 infection, the use of drugs and the aging processes in the era of HAART. A few reports suggested that neuronal α7 nAChR could be activated by HIV-1 virulence factors (e.g., gp120) and related comorbidity factors (e.g., METH) [17, 41–43]. Recent studies show that stimulation of nAChRs impairs the host defense against various microbial infections [15, 44, 45]. The underlying mechanisms of these multiple comorbidities, however, are largely unknown. We have proposed that the HIV-1-related multiple CNS comorbidities are driven by a common set of genes and pathways regulated by α7 nAChR through conveying signaling information from the channel Ca2+ current to IKK/NF-κB activation cascade, leading to upregulation of the proinflammatory factors and enhancement of HIV virotoxins- and comorbidity factors-mediated pathogenicities [15]. It has been suggested that a7 nAChR-mediated NF-kB signaling may be involved in regulation of both the molecular (UCHL1 and S100B) and cellular (cBMEC shedding) biomarkers during various CNS disorders [15]. Using gene knockout mice, we have demonstrated that α7 nAChR plays a detrimental role in the host defense against CNS inflammation caused by microbial (e.g., meningitic pathogens and gp120) and non-microbial factors (e.g., nicotine and METH) [15, 45]. Regulation of intracellular calcium by α7 nAChR can lead to activation of signal transduction pathways, including ERK1/2 and calmodulin-dependent kinase II [44, 45]. In the current study, we demonstrated that CNS disorders induced by *C. neoformans* and HIV-1 associated comorbidity factors could be significantly inhibited by chemical and genetic blockages of α7 nAChR. These new findings concur with our previous studies that gp41 is one of the primary neurotoxins responsible for NeuroAIDS, which significantly enhance meningitic pathogenicities of *C. neoformans* [10, 11]. Furthermore, these pathogenicities are enhanced by METH, one of the commonly abused drugs in patients infected with HIV-1, in a manner dependent on α7 nAChR.

The NF-κB pathway is being increasingly recognized as a good signaling paradigm for molecular biomedicine [46]. This pathway, which is the master regulator of the innate immunity, plays important roles in maintaining cell homeostasis/ differentiation, and regulating the host response to microbial infections [26]. There are five different members in the NF-κB protein family, including p65/RelA, c-Rel, RelB, NF-κB2/p52, and NF-κB1/p50, and sharing a Rel homology domain that mediates DNA binding and dimerization [26]. In resting cells, NF-κB is trapped in the cytoplasm by inhibitory IκB proteins. The NF-κB activation process is induced by phosphorylation of serine residues on the IκB proteins, which are then subjected to ubiquitination and proteasomal degradation. Deregulated activity of this pathway has been linked to the progression of a number of human diseases, including cancers, HIV-1 infection and substance abuse disorders [46–51]. NF-κB activation has been shown to contribute to comorbidity between gp120 and cocaine in neurons [50] or METH in astrocytes [51]. NF-κB inhibitors have been found to reduce the CNS inflammation [52]. Our study demonstrated that *C. neoformans* and gp41-I90 could induce cytoplasmic activation and nuclear translocation of NF-κB in HBMEC through phosphorylation of the p65 subunit at serine 536. gp41-I90- and METH-induced inflammatory response is significantly blocked by inhibitors of α7 nAChR (MLA) and NF-κB (CAPE). These findings suggest that α7 nAChR is required for the modulation of NF-κB activation, which may play an important role in the pathogenesis of CNS comorbidities caused by HIV-1 virotoxins (e.g., gp41) and related factors (e.g., *C. neoformans* and METH). The mechanisms underlying these comorbidities remain to be elusive. It is likely that a common set of genes and pathways regulated by the common receptor α7 nAChR for these comorbidity factors through conveying signaling information from the channel Ca2+ current to IKK/NF-κB activation cascade, leading to the balanced regulation of the proinflammatory and anti-inflammatory factors since the NF-κB signal transduction pathway is the master regulator of the innate immunity.

As the age population increases and HIV-1 infection increases in older population, the incidence of NeuroAIDS becomes higher and accelerated among the aging populations. Likewise, in *C. neoformans* infection, age ≥60 year-old is a significant predictor of the mortality [36, 37]. The incidence of HIV-1 associated neurocognitive disorders (HAND) also increases with age. Current projections suggest that more than 50 % of HIV + patients in the United States will be over 50 years old by 2015 [53]. With advancing age, HIV + patients may potentially develop other neurodegenerative disorders, including Alzheimer's disease. Thus, aging becomes an increasing health concern for these diseases currently. Aging is a complex process, derived from a variety of different mechanisms. Recent studies have suggested that one of the potential mechanisms is the activation of the NF-κB signaling, resulting in chronic inflammatory responses known as the senescence-associated secretory phenotype (SASP) [54–56]. Further, hyper-activation of NF-κB by HTLV-1 Tax can induce cellular senescence [57]. It has been proven that both genetic and pharmacological inhibition of NF-κB signaling prevents age associated features in the animal models, significantly extending their longevity [58]. To test whether and how the α7 nAChR-regulated NF-κB signaling is linked to the pathogenesis and senescence of HIV-1 gp41-I90, METH and *C. neoformans* invasion, we have used the neuronal markers, fluoro-Jade B (FJB) [28, 29] and NeuN [30], to examine the infected mouse brains. The enhancement of FJB signals indicates the increase of neuronal degeneration. On the other hand, the disappearance of the NeuN signals around the cystic lesion suggests the loss of neuron cells in the damaged area. Results from this experiment have provided preliminary support for the hypothesis that the aged mice are more susceptible to pathogen insults, and the vulnerability can be reflected from more brain neuronal damages and the loss of the BBB integrity. BBB dysfunction has been implicated as a crucial event in the development of several aging-related CNS disorders, including Alzheimer disease, Parkinson disease, multiple sclerosis, and HAND [59]. Furthermore, gp41-I90- and METH-induced senescence in HBMECs and astrocytes could be very efficiently blocked by the α7 antagonist MLA, suggesting that α7 nAChR is essential for this pathogenic process.

## Conclusions

Taken together, the major finding of the present report is that both chemical and genetic blockages of α7 nAChR are protective against *C. neoformans*- and HIV-1 associated comorbidity factors-induced BBB injury and CNS disorders by down-regulation of cBMEC shedding, monocyte recuitment, NF-κB signaling, senescence and neuronal inflammation. Further insight into how *C. neoformans* and HIV-1 associated comorbidity factors utilize the host cholinergic α7 nAChR pathway to augment their virulence capacity will advance our understanding of the pathogenesis and therapeutics of CNS disorders caused by multiple comorbidities.

## Abbreviations

AIDS: Acquired immune deficiency syndrome
α7 nAChR: Alpha7 nicotinic acetylcholine receptor
BBB: Blood-brain barrier
BMEC: Brain microvascular endothelial Cells
CAPE: Caffeic acid phenethyl ester
cBMEC: Circulating BMEC
CEC: Circulating endothelial cells
CNS: Central nervous system
DAPI: 4′,6-diamidino-2-phenylindole
HAND: HIV-associated neurocognitive disorders
FJB: Fluoro-Jade B
gp41: Glycoprotein 41
gp41-I90: Gp41 ectodomain peptide (gp41-I90)
HBMEC: Human BMEC
HIV-1: Human immunodeficiency virus-1
HTLV-1: Human T-cell lymphotropic virus type 1
ICAM-1: Intercellular adhesion molecule 1
KO: Knockout
METH: Methamphetamine
MLA: Methyllycaconitine
SAHF: Senescence associated heterochromatin formation
UEA-I: Ulex europaeus I
WT: Wildtype
